# Collateral consequences of COVID-19 for people on probation and parole

**DOI:** 10.1186/s40352-023-00221-0

**Published:** 2023-03-30

**Authors:** Katherine LeMasters, Angela Benson, Christopher Corsi, Taylor Krajewski, Kapriskie Seide, Lauren Brinkley-Rubinstein, Kathryn Nowotny

**Affiliations:** 1 Department of Epidemiology, Gillings School of Global Public Health, University of North Carolina at Chapel Hill, McGavran- Greenberg Hall, CB# 7435, Chapel Hill, NC 27599 USA; 2 School of Social Medicine, Center for Health Equity Research, University of North Carolina at Chapel Hill, Chapel Hill, NC USA; 3 Department of Biostatistics, Gillings School of Global Public Health, University of North Carolina at Chapel Hill, Chapel Hill, NC USA; 4 Center for AIDS Research, School of Medicine, University of North Carolina at Chapel Hill, Chapel Hill, NC USA; 5 Department of Sociology, University of Miami, Miami, FL USA

**Keywords:** COVID-19, Community supervision, Probation, Parole, Collateral consequences

## Abstract

**Background:**

While the severe detrimental impact of COVID-19 on incarcerated people is well known, little is known about the experience of COVID-19 on those on community supervision. Our objective was to better understand the experience of the COVID-19 pandemic and its collateral consequences for those on community supervision (e.g., probation, parole). Beginning in December 2020, we conducted 185 phone surveys about COVID-19 with participants in The Southern Pre-Exposure Prophylaxis (PrEP) Study across its three sites - Florida, Kentucky, and North Carolina. We conducted rapid assessment interviews with both closed- and open-ended questions. We calculated descriptive statistics for close-ended questions and conducted a content analysis for open-ended questions.

**Results:**

The COVID-19 pandemic affected those on community supervision through their experiences in the community and while incarcerated with over one-quarter of participants being reincarcerated during this time. In addition to many (128/185) experiencing COVID-19 symptoms, about half (85/185) of participants reported a diagnosis in their network with 16 of those participants losing loved ones to the pandemic. Participants experienced disruptions to their social network, healthcare, and livelihoods. Though many maintained their support systems, others felt isolated and depressed. Experiences during COVID-19 exacerbated difficulties already faced by those with criminal involvement.

**Conclusions:**

The public health community must recognize those experiencing probation and parole, not only those housed in carceral facilities, as disproportionately impacted by the COVID-19 pandemic. We must tailor programs and services to meet their needs.

## Background

The ongoing COVID-19 pandemic and epidemic of mass incarceration are closely intertwined in the United States (US), with the US having a disproportionate number of COVID-19 cases and individuals involved in its criminal legal (CL) system [1, 2]. Both COVID-19 infection and CL involvement are not randomly distributed, with Black and Hispanic people with low education levels who live in historically segregated and disinvested communities being the most likely to be involved in the CL system and to suffer from COVID-19 [3]–[5]. The harms of COVID-19 are clear within prison and jail walls where social distancing is not possible, personal protective equipment is inconsistently provided and enforced, and testing is infrequent [6, 7]. Therefore, the majority of research on COVID-19 among those involved in the CL system has been focused within carceral facilities that have experienced high COVID-19 case rates [8, 9].

Therefore, there remains a need to understand COVID-19 risk and experiences among those under CL supervision living in the community. People under community supervision (e.g., probation, parole) make up almost two-thirds of those involved in the CL system and also face an increased risk of COVID-19 [10, 11]. First, jail-community cycling has led to heightened COVID-19 community transmission [12]. As mass incarceration is concentrated in disproportionately Black, low-income communities, individuals released from jail on to probation return to the same communities, disproportionately increasing COVID-19 risk. Second, 45% of prison admissions are due to violations of parole and probation, so individuals coming from communities with high COVID-19 are also more likely to be exposed to prison or jail conditions [13]. Third, as probation is increasingly viewed as a positive alternative to incarceration and some prisons and jails decreased their population during the COVID-19 pandemic [14], there is a need to turn attention towards the health of those on community supervision. The population under community supervision is an aging population [15] that disproportionately includes those living in non-carceral institutional settings (e.g., transitional housing ) or experiencing homelessness [16], and is largely made up of Black and Hispanic people [17], all of whom experience an increased risk of COVID-19 [18]. Furthermore, this population experienced poor health and low access to healthcare services prior to the COVID-19 pandemic, further increasing their risk of severe infection and delays in care [19]. Given these many risk factors, there is a need to better understand how the pandemic impacted this often-overlooked population that straddles both the CL system and local communities.

The COVID-19 pandemic also likely had disproportionate *collateral* effects on this population. As individuals re-integrate into communities after leaving incarceration, they have many competing priorities and unmet basic needs (e.g., food, shelter, medical appointments) [20] that were likely heightened during COVID-19. Individuals also frequently lose social networks while incarcerated, likely leading to increased isolation and less support during a time when many already faced intense loneliness [21]. This population also has low pre-existing levels of institutional and medical trust due to historic dehumanization and mistreatment in CL settings and increasingly policed healthcare settings in communities [22]–[24]. Data from early in the COVID-19 pandemic showed that this held true with 15% of women involved in the CL system stating that they did not trust any information on COVID-19 [25]. Furthermore, rules and regulations associated with probation, parole, and re-incarceration have constantly changed during the COVID-19 pandemic, as courts and probation and parole offices are intermittently closed and prisons and jails have attempted to lower admissions, producing an unpredictable environment, and much of community supervision has transitioned to remote settings [26]–[30].

The COVID-19 pandemic has severely impacted those incarcerated in prisons and jails in the US, but little is known from the perspective of those on probation and parole in the US about how this pandemic has affected them. Understanding how the pandemic has affected people on community supervision – from their perspective – will allow the public health and medical communities to better advocate for social services and medical care for this often overlooked population. Our research question asked how those on community supervision in the US experienced the COVID-19 pandemic and what collateral consequences they faced.

## Methods

SPECS (The Southern Pre-Exposure Prophylaxis (PrEP) Study) is an 18-month prospective cohort study designed to respond to and close the knowledge gap regarding PrEP, a widely available, daily medication efficacious in preventing HIV, and those involved in the CL system in three diverse southern settings: Florida, Kentucky, and North Carolina [31]. HIV prevalence among CL-involved individuals is five times that of the general population and following release from incarceration, individuals face multi-level barriers to HIV prevention [32]. The goal of SPECS is to investigate barriers and facilitators for PrEP initiation and sustained use among CL-involved adults, building a foundation for PrEP interventions for this underserved population. In Fall of 2019, SPECS began recruitment and after the COVID-19 pandemic began in Spring of 2020, the SPECS team added an additional COVID-19 survey. Beginning in December 2020, we conducted 185 phone surveys about COVID-19 with SPECS participants across these three sites. Participants were compensated $30 for the phone surveys. The majority ofsurveys (94%) were conducted before April 2021 (i.e., before COVID-19 vaccinations were widely available).

Surveys included both closed-ended and open-ended questions, which assessed participants’ COVID-19 experienced and collateral consequences from the pandemic. The close-ended questions concerning COVID-19 clinical information were modeled after the COVID-19 survey developed for the Multicenter AIDS Cohort Study and Women’s Interagency HIV Study early in the COVID-19 pandemic (D’Souza et al., 2020; Trotter et al., 2001).We also included the Pandemic Stress Index developed by researchers in 2020 [33]. The majority of close-ended questions were asked in a yes/no format and are summarized in the results section. Open-ended questions asked participants about change in communication with parole and probation officers as well as disruptions to community supervision and their day-to-day life overall. The surveys were not audio-recorded therefore responses to open-ended questions were not recorded verbatim. While contemporaneous notetaking did not capture verbatim responses, interviewers filtered responses in the transcription process using respondents’ own words whenever possible. The survey contained seven sub-sections: (i.e., health history during the COVID-19 pandemic, COVID-19 precautions taken, how COVID-19 affected their daily life, how COVID-19 affected access to medical care, mental health, social support, response to policy changes during COVID-19). All data were collected via a REDCap form. The survey took approximately 30 min to complete. This study was reviewed and approved by the institutional review board at the University of North Carolina at Chapel Hill (18–2466).

Descriptive statistics were for the quantitative data was generated using Windows SAS version 9.4 (Cary,NC). For analysis of open-ended questions, we drew inspiration from the analytic approach of DeHart, Lynch, Belknap, et al. to analyze notes taken by interviewers, which served as the response to open-ended questions [34]. We coded the notes to summarize pertinent information and identify specific qualitative exemplars that illustrate findings revealed in the quantitative analysis of participants’ COVID-19 experiences and collateral consequences of the pandemic. We presented these references using a third-person perspective to emphasize that they are not direct quotes.

## Results

Between June 2019 and March 2020, SPECS enrolled 227 participants, 185 (81%) of which completed a COVID-19 survey between December 2020 and August 2021. The 185 participants were enrolled at three different sites, 47% in Kentucky, 35% in Florida, and 19% in North Carolina. About 17% identified as LGBTQ; median age was 34 (IQR 27–43) years and 33% were female; 38% were White non-Hispanic, 38% Black non-Hispanic, 17% Hispanic/Latinx, and 7% other or unknown. About half (46%) of participants were on parole/post-release supervision and 54% were on probation.

## COVID-19 experiences

Over two-thirds (128/185; 69.2%) of participants experienced COVID-19 symptoms, though there was a high amount of uncertainty as to whether COVID-19 caused these symptoms. Around two-thirds (114/185; 61.6%) had been tested for COVID-19, and participants had varied reactions to getting tested and perspectives on acquiring COVID-19. Of 185 respondents, five had a confirmed COVID-19 case and one was hospitalized with COVID-19. Among participants who hadn’t been tested but reported symptoms consistent with COVID-19, reasons for going untested ranged from resignation (e.g., if they get it, they get it) to low risk perception (e.g., they did not think they could get it).

For others, practical limitations prevented them from getting tested, particularly due to barriers posed by the CL system. CL re-involvement varied in this time, with 28% (51/185) of participants being reincarcerated between January 2020 and time of survey completion. One participant described being in a treatment facility that was locked down. Another referenced their time in incarceration, noting that, although they believe they may have caught COVID-19 while incarcerated, they had no way of being certain because they were not able to get tested.

### Impact on social networks

In answers to open-ended questions, participants expressed worry about friends, family, and household members who were young, elderly, immune-compromised, CL-involved, or struggling with addiction. About half (85/185; 46.0%) of respondents said that at least one friend or family member had been diagnosed with COVID-19 with 31% (26/85) of those participants noting a severe COVID-19 infection in their social network that resulted in hospitalization and 19% (16/85) experiencing the death of a loved one. These respondents reported losing friends and family members, especially older relatives, to COVID-19 infection. They feared experiencing more losses or infecting remaining members of their social circles, particularly when these close circles included individuals who may be more likely to have severe complications or die from the virus (e.g., elderly relatives). When asked about the impact of the pandemic on her life overall, one participant reflected on how acutely the virus had impacted her family:

She had to change her whole entire life, the way she works. Her family changed, it killed almost the entire older generation of her family. They were not able to see each other at any holidays. They were avoiding each other and they still died. So, it’s impacted them a lot.

Thus, the COVID-19 pandemic both directly affected her social network through multiple older family members dying from COVID-19 and indirectly affected her network by changing her way of life and who she was able to see regularly.

### Disruptions in healthcare access

Beyond acquiring COVID-19, the pandemic affected peoples’ ability to obtain physical and mental healthcare. One-quarter of participants (46/185, 24.9%) stated that they were unable to seek physical healthcare. Of these individuals, 72% (33/46) stated that the medical facilities were closed, 22% (10/46) lacked transportation to get to the appointment, and 22% (10/46) lacked access to telehealth services being offered. Sixteen of the 185 respondents (9%) were unable to access their regular medication and twenty-one (11%) could not afford healthcare - most due to losing insurance during the pandemic. Of the 31% (57/185) that received mental healthcare, 19 had their care disrupted. Of the 32% (59/185) that receive substance use treatment, about half (28/59; 47.5%) had their care disrupted. About one fifth (35/185; 18.9%) of respondents receive both mental healthcare and substance use treatment, with 7 of them (20%) having both types of care disrupted. Yet, in the midst of these disruptions, individuals stated that the pandemic had opened their eyes to the need to focus on their health and take their health into their own hands.

### Precautions taken and reactions to the pandemic

Most of the 185 participants (79%) who completed the COVID-19 survey said that they were taking the pandemic seriously. Almost all participants attested to trying to prevent COVID-19 by staying at home (89%), social distancing (94%), wearing a mask in public (98%), and practicing hand hygiene (97%). Over half of participants said that they socially distanced themselves to protect someone in their household. Multiple participants said that they took precautions because they were scared for themselves, their children, and their families. For example, one individual said that while he did not want to practice social distancing, he did it to protect his grandmother. Another said that his employers enforced masking, which resulted in him being more stringent.

However, taking precautions was made difficult during stays of incarceration, where it was quite difficult to social distance and receive masks or any personal protective equipment. Those experiencing incarceration duringthe pandemic also noted that staff did not wear masks but consistently came and went from the community, likely serving as the vector for COVID-19. Individuals who tested positive for COVID-19 were also housed together in a single unit.

## COVID-19 collateral consequences

### Economic impacts

COVID-19 has had collateral consequences on individuals’ social and economic wellbeing. Sixty-seven individuals lost a source of income, with sources of income being a pre-existing stressor for many with recent CL involvement. The majority of individuals that lost their income were laid off of work or their workplace closed. Furthermore, individuals mentioned having a difficult time maintaining full-time work hours due to working in bars and restaurants, which had many COVID-19 restrictions. This made it particularly difficult to meet basic needs (e.g., utilities, rent). The pandemic and their CL involvement made it multiplicatively difficult to find new employment with some beginning work in informal employment sectors. One participant expanded on this struggle:

​She was working at a job and then was exposed to COVID. She had to quarantine and get tested, and at the end of that entire process she came back to her place of employment and they said they ran out of hours for her. She has not been able to find work since, and she thinks a lot of that does have to do with her record. Right now, the person who owns the place she lives in has asked her and her husband to move out, so things have been really stressful - she does not know what the shelter situation would even look like during COVID.

For this participant, economic difficulties were compounded by COVID-19 exposure, the stigma of CL-involvement, and a tenuous living situation. These types of burdens also take significant tolls on mental health.

### Mental Health concerns and social support

Many individuals became increasingly fearful and worried about their circumstances, often feeling isolated and lacking social support. About 60% (109/185) of participants noted that they were worried about their family. Some also lacked consistent internet connectivity (8/185; 4.3%) and or a smartphone (12/185; 6.5%), a potentially important component to staying connected with social support during the pandemic. Multiple participants said that they experienced depression and loneliness during this time, feeling overwhelmed by the world shutting down.Survey notes indicated heightened feelings of anxiety, specifically around touching surfaces, being around other people, or leaving the house.

Individuals expressed that the pandemic made them feel stressed, hopeless, confused, nervous, worried and concerned about restrictions, their health, their children, and the unknown. To cope, some found solace in their social network, feeling gratitude and renewed appreciation for the people in their lives. However, 14 (8%) individuals stated that they had no one to rely on for support and nine (5%) individuals were dissatisfied with their support. The majority of participants (72%) said that they had two or more people to rely on for support and were satisfied or very satisfied with the support they received from others. About one third (58/185; 31.4%) of participants did not report feeling lonely at all through this portion of the pandemic. During one survey, a participant explained that COVID had made it extremely hard to do anything, including working or living, demonstrating how challenging the pandemic has been for them. However, when they were asked how the outbreak had affected them generally, their response centered on social support: it made them value the people in their life.However, 14 (8%) individuals stated that they had no one to rely on for support and nine (5%) individuals were dissatisfied with their support.

While rules and regulations associated with community supervision (e.g., regular fees, mandatory in-person meetings) were relaxed during the COVID-19 pandemic, those that remained in place were often confusing. Several respondents indicated that these changes were positive, noting how probation officers would complete meetings over the phone or by coming to their homes, and how this reduced the burden of community supervision. One participant explained these changes were humanizing:

He found supervision to be much more lenient, which was fantastic. That’s why COVID has helped, it makes the law lay off of you. They started treating drug addicts like human beings. They started prioritizing who they lock up, and that’s a good thing.

However, a few individuals had inconsistent experiences with their probation and parole officers during this time, which contributed to their stress. Among confusion around how to contact closed offices, meet with officers during lockdown, and complete classes or community service requirements, some participants reported experiencing extended supervision time, or violations and arrests, which in turn placed them in carceral facilities at higher risk of exposure.

Individuals who experienced incarceration during the pandemic indicated the heightened restrictions also took a significant toll on their mental health. When asked about COVID-19 and incarceration, one participant reflected on his experiences with solitary confinement:

Right now, he said, everyone - when they get locked up and go into the jail, they have to do two weeks of the hardest solitary confinement you have ever seen. He asked if we knew what solitary confinement does to a person − 23.5 hours of solitary confinement a day, and some of these people are innocent. A lot of people do not know what that feels like. No visits either - the visits are video visits, there is no contact, not even a window, they stay in their cells. He asked if there is anywhere in the world where this makes sense.

In total, eight participants reported experiencing strict fourteen-day solitary confinement as medical quarantine, which caused significant distress.

### Institutional trust

Many participants lacked institutional trust and held conspiracy beliefs. Over one-third (69/185; 37.3%) stated that they did not trust that the government was doing all it could to prevent the spread of COVID-19, 29% (54/185) did not trust information from the Centers for Disease Control and Prevention (CDC) on COVID-19, and 23% (43/185) did not trust information from the state health department about COVID-19. In their answers to open-ended questions, participants expressed varying levels of trust in different institutions (e.g., jail, government, media, and medical institution) vis-à-vis the pandemic. This lack of trust was amplified due to information from the CDC and government being counter to what was said in the CL system. One individual noted that while the state government recommended staying home, their probation officer wanted them to come in-person, and when they did not, they were re-incarcerated. Furthermore, one participant identified quarantine in jail as a burden placed on those with CL involvement not shared by officers.

## Discussion

The key findings of this study were that the COVID-19 pandemic has deeply affected those on community supervision, both through their experiences in the community and while incarcerated. Many individuals experienced COVID-19 symptoms and lost loved ones to the pandemic. They also experienced disruptions to their social network, healthcare, and livelihoods. Though many maintained their support systems, others felt isolated and depressed. Experiences during COVID-19 have exacerbated well-documented difficulties already faced by those with CL involvement: few employment opportunities - often in service industries, difficulties in meeting basic needs, and low trust in institutions [20, 24].

Many participants experienced reincarceration during this time and were keenly aware of the COVID-19 risk posed within carceral facilities [12]. Before the COVID-19 pandemic, in 2019, over 153,000 individuals were re-incarcerated in the US for non-criminal violations of probation or parole. While states claim to have reduced their incarcerated population, national reports and reports from SPECS participants indicate that these efforts often fell short [10, 26, 35]. Furthermore, carceral staff’s disregard for COVID-19 precautions was both frustrating and jolting, often at odds with guidance individuals were hearing from other government entities such as the state health department and the CDC. As a result, individuals were often confused by the guidance and what precautions they should be taking. Given that state health departments often do not collaborate with state Departments of Correction, including during the COVID-19 pandemic, these findings are not surprising but should serve as an additional call to action for collaboration [36]. Additionally, guidelines that do exist for carceral staff do not extend to probation and parole officers, emphasizing how this population is often overlooked in public policy.

We have three primary recommendations for community supervision and for the public health community. First, g iven the many difficulties faced by those on community supervision that are exacerbated by COVID-19, it is critical that information on COVID-19 itself and efforts to mitigate COVID-19’s collateral consequences (e.g., rental assistance) are made available to this population. This information must be placed in areas those on probation and parole often access (e.g., transitional housing, substance use treatment programs, disability services) and program staff must be well-acquainted with how to assist individuals in accessing these services. Second, peer navigator services have proven successful at connecting previously incarcerated individuals with health services and should be applied during the COVID-19 pandemic as well in a broader sense to connect individuals with services [37]. Third, community supervision requirements (e.g., monthly fees, mandatory visits) should be canceled, as they are particularly burdensome during this time, and updated guidance should be clear, as the constant changes were confusing and stress-inducing for participants [38]. Staff members in probation and parole offices should also closely follow COVID-19 best practices and adhere to guidelines from Departments of Health.

There are multiple limitations and areas for future research. First, as this was primarily a quantitative survey with optional open-ended questions, the field notes are not representative of the entire sample and are not quotes. We are unable to analyze, for example, how peoples’ experiences varied by geographic location. Geography serves as important context both for probation and parole and for the COVID-19 pandemic. Second, given the ever-changing nature of the COVID-19 pandemic, the prevalence of COVID-19 symptoms and collateral consequences on individuals’ lives are likely quickly changing. We hope that future research continues to understand the impact of the pandemic on this population.

## Conclusions

COVID-19 poses a high burden for those involved in the criminal legal system, including those on community supervision. The public health community must recognize those experiencing probation and parole as disproportionately impacted by this ongoing pandemic and must tailor programs and services to meet their needs.

## Data Availability

The datasets used and/or analyzed during the current study are available from the corresponding author on reasonable request.

## List of abbreviations

US: United States
CL: Criminal Legal
PrEP: Pre-Exposure Prophylaxis
CDC: Centers for Disease Control and Prevention
